# Impact of Timely Public Health Measures on Kidney Transplantation in Austria during the SARS-CoV-2 Outbreak—A Nationwide Analysis

**DOI:** 10.3390/jcm9113465

**Published:** 2020-10-28

**Authors:** Bruno Watschinger, Clara Watschinger, Roman Reindl-Schwaighofer, Elias L. Meyer, Andras T. Deak, Tamara Hammer, Manfred Eigner, Hannelore Sprenger-Mähr, Stefan Schneeberger, Daniel Cejka, Gert Mayer, Rainer Oberbauer, Alexander R. Rosenkranz, Julia Kerschbaum

**Affiliations:** 1 Department of Nephrology, Medical University of Vienna, 1090 Vienna, Austria; clarawatschinger@gmail.com (C.W.); roman.reindl-schwaighofer@meduniwien.ac.at (R.R.-S.); rainer.oberbauer@meduniwien.ac.at (R.O.); 2 Section for Medical Statistics, Center for Medical Statistics, Informatics, and Intelligent Systems, Medical University of Vienna, 1090 Vienna, Austria; elias.meyer@meduniwien.ac.at; 3 Division of Nephrology, Department of Internal Medicine, Medical University of Graz, 8036 Graz, Austria; andras.deak@medunigraz.at (A.T.D.); Alexander.Rosenkranz@medunigraz.at (A.R.R.); 4 Department of Medicine III: Nephrology, Transplantation Medicine, Rheumatology, Geriatrics, Ordensklinikum Linz Elisabethinen, 4010 Linz, Austria; Tamara.Hammer@ordensklinikum.at (T.H.); Daniel.Cejka@ordensklinikum.at (D.C.); 5 Department of Medicine I, Klinik Favoriten, 1100 Vienna, Austria; manfred.eigner@gesundheitsverbund.at; 6 Landeskrankenhaus Feldkirch Abteilung für Innere Medizin III, Nephrologie und Dialyse, 6807 Feldkirch, Austria; Hannelore.Sprenger-Maehr@lkhf.at; 7 Department of Visceral, Transplant and Thoracic Surgery, Medical University of Innsbruck, 6020 Innsbruck, Austria; stefan.schneeberger@i-med.ac.at; 8 Department of Internal Medicine IV, Nephrology and Hypertension, Medical University Innsbruck, 6020 Innsbruck, Austria; gert.mayer@i-med.ac.at (G.M.); Julia.Kerschbaum@i-med.ac.at (J.K.); 9 Austrian Dialysis and Transplant Registry, Department of Internal Medicine IV, Nephrology and Hypertension, Medical University of Innsbruck, 6020 Innsbruck, Austria

**Keywords:** SARS-CoV-2, COVID-19, kidney transplantation, public health measures, patient survival

## Abstract

SARS-CoV-2 led to considerable morbidity/mortality worldwide and tremendously impacted on daily life. Strict lockdown measures were implemented early to contain the viral outbreak in Austria. Massive changes in organizational structures of healthcare facilities followed with unclear implications on the care of non-COVID-19-affected patients. We studied the nationwide impact of COVID-19 on kidney transplantation in Austria during the first six months of 2020. Concurrent with general lockdown measures, all kidney transplant activity was suspended from 13 March to 9 April. Nevertheless, between January and June, total transplant (*p* = 0.48) and procured donor organ numbers (*p* = 0.6) did not differ significantly from earlier years. Ten (0.18%) of 5512 prevalent Austrian kidney transplant recipients were diagnosed with SARS-CoV-2. The case fatality rate (one death; 10%) in renal transplant patients was less than in other countries but higher than in Austria’s general population (2.4%). We conclude that early and strict general lockdown measures imposed by the government allowed an early, however cautious, re-opening of Austrian transplant programs and played a crucial role for the favorable outcomes of SARS-CoV-2 in Austrian kidney transplant patients. Even though it may be uncertain whether similar results may be obtainable in other countries, the findings may support early intervention strategies during similar episodes in the future.

## 1. Introduction

After SARS-CoV in 2003 and MERS-CoV in 2012, SARS-CoV-2 has emerged as the source for a third rapidly spreading coronavirus outbreak [1]. In the meantime, COVID-19 was declared a global pandemic by the World Health Organization [2] and has led to over 28 million reported cases and over 915,000 deaths worldwide [3]. COVID-19 courses seem to be particularly severe in immune-compromised hosts with greater rates of ICU admissions and death than in the general population (fatality rate ~ 13–28% [4]). Among others, several larger case series reported on clinical courses and outcomes of COVID-19 in kidney transplant recipients in various regions of the world [5,6,7,8,9,10]. Besides the effects on individual patients, the pandemic had a significant impact on organizational aspects of transplantation. Early reports from Italy, France and the US that indicated a rapid decline in transplant numbers [11,12,13] prompted us to study the associations and early implications of the SARS-CoV-2 pandemic on the national kidney transplant activity in Austria** (population 8.9 million), in which transplant numbers per million inhabitants are among the highest in the world [14]. In particular, we were interested to examine if early restrictions promoted by the Austrian government and the good adherence of the Austrian public to these measures may have played a role in the continuation of kidney transplant activity in the first months of 2020.

### SARS-CoV-2 in Austria

On 25 February 2020, the first two cases of SARS-CoV-2 infections were recorded in Innsbruck. During the subsequent days, Austria saw an exponential increase in cases, congruent with increases seen in other countries worldwide. The government promoted strict adherence to personal hygiene as well as social distancing measures, and followed a “stay-at-home testing” strategy (symptomatic patients could call a Health Hotline, that provided medical advice and organized tests at home) to spare hospitals and healthcare facilities to the utmost degree from unnecessary exposure to infected patients. SARS-CoV-2-positive, mildly symptomatic subjects had to home quarantine. Contact tracing went into effect to test contact persons and impose quarantine on them.

By 11 March, the daily new SARS-CoV-2 cases had reached 206 in Austria. Consequently, the government imposed far-reaching restrictions, which were implemented in a stepwise fashion (see Table 1). Hospital capacities were established to prepare for an imminent strain on the health system. Certain hospitals and wards were dedicated preferentially for SARS-CoV-2-infected patients. All non-emergency surgery was cancelled to reserve intensive care (ICU) capability for pending COVID-19 patients. Wherever feasible, outpatient visits were postponed.

These regulations affected kidney transplant programs, as transplantation (with dialysis being an alternative treatment option) was classified as a non-lifesaving, semi-elective surgical procedure that could be postponed to after the resolution of the viral threat. The decision to pause transplant programs on 13 March was corroborated by the uncertainty regarding possible virus transmission from a donor (if not adequately tested) and the unclear effect of intense post-transplant immunosuppression on viral defense mechanisms of the recipient.

## 2. Methods

We retrospectively studied the effects of the pandemic on kidney transplantation and on incident and prevalent transplant recipients in Austria. Transplant activity in the four Austrian transplant centers (Medical Universities of Vienna, Innsbruck and Graz and Ordensklinikum Linz) during the first half year of 2020 was compared to preceding years. Timely correlations with the general governmental regulations and the specifications imposed on organizational structures in Austrian hospitals were analyzed. This included total transplant activity, national organ procurement and issues concerning post-transplant patient care. Austria is part of Eurotransplant (Members: Austria, Belgium, Croatia, Germany, Hungary, Luxembourg, the Netherlands and Slovenia) which is serving a total population of approximately 137 million. Organs are procured and distributed within the member states.

Incident kidney transplants performed in the first half of 2020 (in particular between March and June, following the temporary suspension of transplantation) were evaluated and compared to preceding years (2016–2019).

The clinical course and outcome of all COVID-19-infected kidney transplant recipients, who were reported to the Austrian Dialysis and Transplant Registry (OEDTR), were recorded. All infected patients were followed for a median of 94 days (range 19 to 107 days) until 30 June. The incidence rate of SARS-CoV-2 in Austrian patients was calculated based on the total number of prevalent kidney transplant recipients.

The effect of COVID-19 on post-transplant care was evaluated at the four transplant centers. The weekly outpatient activities for each center were recorded from 1 January to 30 June and compared to the respective weeks in 2019.

The study was approved by the local ethics committee (EK Nr: 1704/2020) and conducted in accordance with the Helsinki Declarations. Total transplant and donor numbers for the periods January–June 2016–2020 were visualized using grouped barplots and boxplots. To compare the average monthly transplant numbers between the years 2016 and 2020, an ANOVA was used. No post hoc tests were conducted. The number of weekly outpatient visits during the first six months of 2019 and 2020 in the four transplant centers was visualized as line graphs and compared by paired *t*-tests for all four centers. *p*-values < 0.05 were considered statistically significant, however, as they serve only descriptive purposes, no multiplicity correction is applied.

## 3. Results

In the light of rapidly rising numbers of SARS-CoV-2 infections throughout Europe and after a thorough discussion between representatives of the four Austrian transplant centers, it was convened on 13 March 2020 to suspend all kidney transplant activity in the country. Daily reports on SARS-CoV-2 case numbers in the Austrian general population were closely monitored. When Austria saw a sustained reduction (118 new cases on 14 April) and the SARS-CoV-2 situation within most Eurotransplant member states was equally easing up, all centers agreed to restart the deceased donor programs. Initially, only low-risk transplants were considered. Antibody induction therapy is generally not used in patients with a lower immunological risk in Austria. In these patients, immunosuppression did not differ from patients transplanted in the pre-COVID period. The first deceased donor transplant after the lockdown was performed in Innsbruck on 9 April. The living donor programs were resumed at different times (Innsbruck 23 April, Vienna 18 May, Linz 1 June, Graz 8 June). Vienna has accepted highly sensitized patients (who usually receive polyclonal antibodies as induction therapy) since 14 May (Innsbruck 6 May, Linz 17 April, Graz 8 June). The protocols for higher-dose immunosuppression (including, e.g., immunadsorption and the use of lymphocyte-depleting agents) were not adapted from pre-COVID times. Pre-transplant evaluations, a prerequisite for being accepted to the waiting list, resumed during mid-April.

### 3.1. Transplant Activity in the Four Austrian Transplant Centers

**Figure 1:**
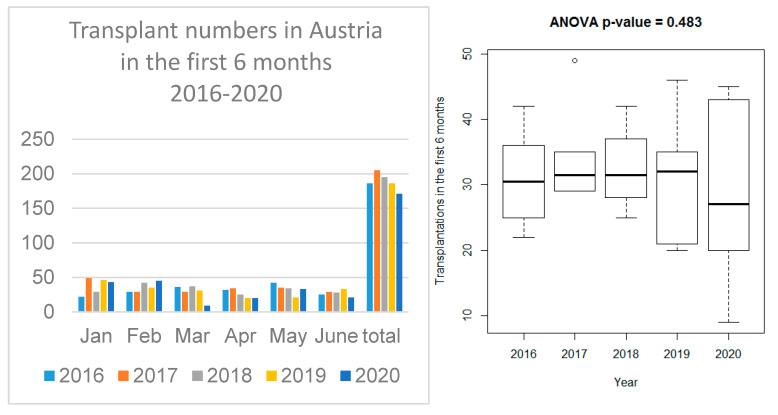
Monthly number of kidney transplants in Austria during the COVID-19 pandemic (January to June 2020) and comparison of the monthly average in previous years by ANOVA (data from the four Austrian transplant centers in Vienna, Innsbruck, Graz and Linz).

The monthly rate of kidney transplants (January to June 2016–2020) at the four centers is shown in Figure 1. Apart from March 2020, when it was decided to temporarily stop the programs and given the variation in the monthly numbers throughout the years, no striking effect of SARS-CoV-2 on the transplant activity is obvious. The average six-month transplantation numbers did not differ significantly from 2016 to 2020 (*p* = 0.483), as shown in Figure 1. The mean transplant numbers (±Stddev) during the first six months of the years 2016–2020 were 31.0 ± 6.7, 34.2 ± 7.1, 32.5 ± 5.8, 31.0 ± 8.8 and 28.5 ± 13.0, respectively.

### 3.2. Deceased Donor Transplantation

From 1 January to 30 June, 151 deceased donor kidney transplants were performed in Austria (62 in Vienna, 59 in Innsbruck, 10 in Graz and 20 in Linz). Until 13 March 2020 (when the transplant activity was intermittently stopped), 84 kidney transplants were performed in Austria (35, 32, 6 and 11 in Vienna, Innsbruck, Graz and Linz, respectively). Following the re-opening of the program on April 9 in Innsbruck, 15 April in Vienna and Linz and 4 May (due to local institutional regulations) in Graz until 30 June, 67 patients received a kidney in Austria (27, 27, 4 and 9 in Vienna, Innsbruck, Graz and Linz, respectively).

### 3.3. Living Donor Transplantation

The living donor transplant programs in Austria were equally paused on 13 March and were resumed at different times in the four centers. Before the lockdown, seven living donor transplants were performed in Innsbruck, five in Vienna and one in Linz. After the lockdown, Innsbruck resumed the living program in April with two transplants (May *n* = 1, and June *n* = 2), and the other centers restarted the evaluation of new donor/recipient pairs on May 18, but did not perform living donor transplants in May. In June, Linz performed one living donor, and in Vienna, an initially re-scheduled transplantation was performed.

### 3.4. Organ Procurement

**Figure 2:**
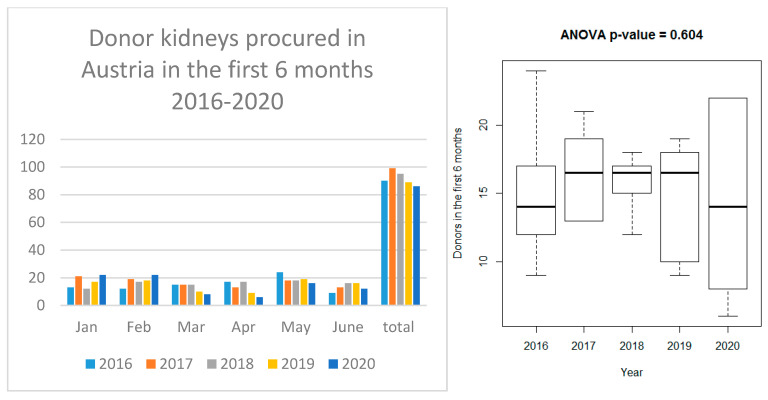
Monthly number of procured kidneys in Austria during the COVID-19 pandemic (January to June 2020) and comparison of the monthly average in previous years by ANOVA (data from the four Austrian transplant centers in Vienna, Innsbruck, Graz and Linz).

The organ procurement rate remained stable in the first half of 2020 (January to June). The average monthly donor numbers did not change significantly from 2016 to 2020 (*p* = 0.604) (Figure 2). The mean donor numbers (±Stddev) during the first six months of the years 2016–2020 were 15.0 ± 4.7, 16.5 ± 3.0, 15.8 ± 2.0, 14.8 ± 3.9 and 14.3 ± 6.3, respectively.

### 3.5. SARS-CoV-2 Infections in Austrian Transplant Patients

**Figure 3:**
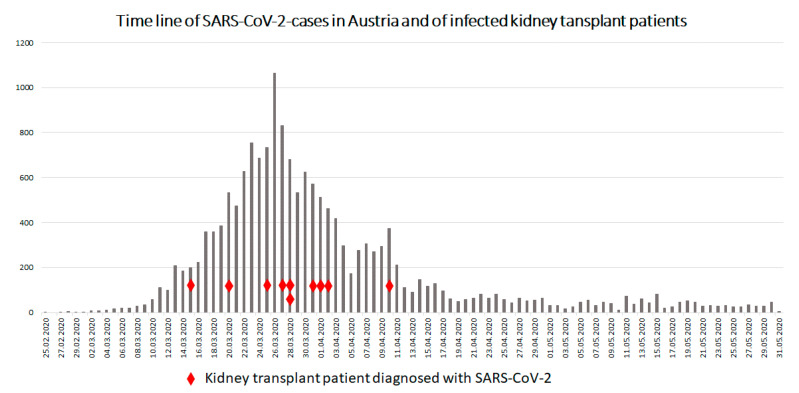
Daily timeline of new SARS-CoV-2 cases in Austria and time of occurrence of SARS-CoV-2 infection in the 10 affected kidney transplant recipients (Source: BMSGPK, Österreichisches COVID-19 Open Data Informationsportal (https://www.data.gv.at/covid-19; accessed on 7 August 2020) and Austrian Dialysis and Transplant Registry).

According to the Austrian Dialysis and Transplantation registry, 5512 kidney transplant recipients were alive at the end of February 2020. From the start of the outbreak until 30 June, ten patients tested positive for SARS-CoV-2. The time of diagnosis of the individual patients in relation to the general Austrian SARS-CoV-2 situation is depicted in Figure 3. Three patients, initially presenting with mild symptoms, were treated on an outpatient basis. Seven patients were admitted to the hospital because of moderate to severe symptoms for a mean of 14.1 days (range 5 to 24 days). Two patients had to be admitted to the ICU. One patient recovered, the other died from COVID-19 pneumonia. Patient details are shown in Table 2. No SARS-CoV-2 infections occurred in patients who were transplanted since March (after the emergence of the virus in Austria).

### 3.6. Post-Transplant Care of Austrian Kidney Recipients

**Figure 4:**
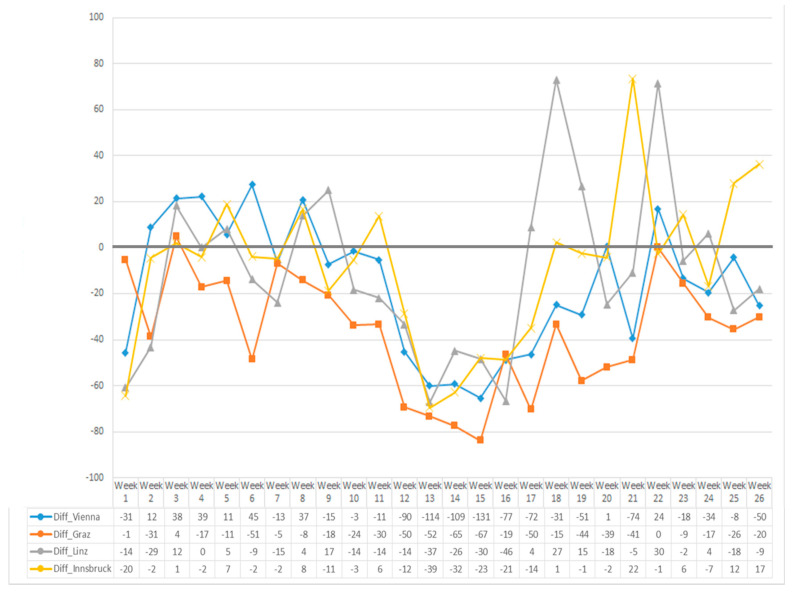
Number of weekly outpatient visits to the kidney transplant clinics at the University of Vienna, Innsbruck, Graz and Linz from January to June 2020 compared to the respective weeks in 2019. The line graph depicts the relative change from 2019 to 2020, and the table shows the changes in absolute numbers. Differences are significant for Vienna and Graz (both *p* < 0.01), but not in Linz and Innsbruck (*p* = 0.069 and *p* = 0.129).

The majority of transplant recipients are followed as outpatients by their transplant center (as there are practically no transplant physicians practicing outside hospitals in Austria), amounting to considerable numbers of patients treated in outpatient clinics. In 2019, the average number of patients seen per week in the centers were Vienna *n* = 173, Innsbruck *n* = 45, Graz *n* = 71 and Linz *n* = 55. During the COVID-19-induced lockdown, Austrian hospitals were requested to limit outpatient contacts to emergency visits only. Therefore, non-essential visits to the transplant outpatient clinics were actively postponed. The centers remained accessible for emergencies and by phone or video consultations, whenever necessary. Weekly outpatient numbers differed significantly between 2019 and 2020 in Vienna and Graz (both *p* < 0.01), but not in Linz and Innsbruck (*p* = 0.069 and *p* = 0.129), as shown in Figure 4.

## 4. Discussion

SARS-CoV-2 and its associated disease, COVID-19, have led to widespread consequences for all aspects of daily life, as well as for national economic and healthcare systems. The official response to the viral threat differed significantly between countries [15,16]. The exponential increase in SARS-CoV-2 infected patients led to an early intervention by the Austrian government starting on March 16, when the number of new daily cases had reached 206 (Table 1). These orders came much earlier than in other countries that were hit by the pandemic. The general Austrian response to the coronavirus was considered very good in a recent study among 21 OECD countries (considering the number of performed tests, provision of non-COVID-19 healthcare and the number of above average excess deaths) [16]. Early, effective interventions and lockdown measurements have been shown to be fundamental in minimizing the COVID-19 death rate in European countries [15] and physical distancing, face masks and contact tracing have been proven effective [17].

In fear that the hospital and intensive care capacity could be overwhelmed by the ongoing pandemic, Austrian hospitals started to take precautions, which included the provision of treatment capacity for SARS-CoV-2-infected patients. All elective surgical procedures were cancelled by 12 March. Simultaneously, all planned non-essential outpatient visits were postponed. Despite missing empirical evidence at the time [18], it was convened by the Austrian transplant centers to temporarily hold the kidney transplant programs on 13 March, as dialysis can serve as an alternative treatment option for end-stage renal disease patients. Many other centers around the world equally interrupted their living and deceased donor programs [11,12,19,20,21,22,23,24].

A reason for pausing the transplant activity was the unclear situation regarding the prevalence of SARS-CoV-2 in other Eurotransplant member states that would be part of the organ exchange program. Furthermore, the available test capacities in different countries were unknown and it was uncertain whether all potential organ donors can be adequately tested for SARS-CoV-2. Thus, a potential virus transmission from the donor to the recipient could not be excluded and remained another obstacle to a safe organ retrieval. Travel restrictions and the grounding of air travel made organ transport virtually impossible or would have led to prolonged cold ischemic times of unacceptable lengths in most instances. In addition, it was unforeseeable to which degree of risk an individual patient would be exposed by the typically intensive immunosuppression immediately post-transplant.

Contrary to the expectations, the four-week intermission until mid-April did not have a significant impact on the mean monthly number of transplants in Austria, when compared to the first six months in preceding years. To our knowledge, this is the first complete report at a national level on the effect of SARS-CoV-2 in kidney transplantation. Due to the current lack of equally concise national transplant data during the early months of the pandemic elsewhere, it is yet beyond the scope of our manuscript to compare the Austrian data with other nations. A similar advantageous recovery of transplant numbers, however, was reported on a regional basis for Cantabria, Spain [25]. This is in contrast to various datasets reported from Italy, France and the United States, where the pandemic led to a significant reduction in kidney transplantations [11,12,13]. The lack of ICU capacity makes it difficult to re-start the transplant programs in Italy [11]. In Austria, the ICU capacity could be well preserved. Other than in Spain or Italy [20,26], the number of procured donors did not decline in the first half of 2020.

The reason for these advantageous results in Austria may be the favorable general virus situation in the country, which was critically influenced by early testing and home care strategies and the restrictions implemented by the government. COVID-19 patients, once identified, were admitted to designated COVID hospitals or wards according to the national pandemic plan. This strategy helped to keep transplant centers COVID-free during the observation period. This successful way helped our decision to re-start the transplant programs early after freezing them and to provide organs for our patients again. Thus, SARS-CoV-2 showed only a mitigated effect on the Austrian transplant activity (Figure 1 and Figure 2). Admittedly, we cannot rule out that without COVID-19 the numbers of performed transplants could have been higher. It remains unknown how many transplant opportunities were missed in the respective “transplant-lockdown period”. In the UK, over 1600 missed transplant opportunities were calculated for a six-month period starting on 5 March 2020 with an expected increase in the number of patients on the waiting list [27]. The patients’ reactions to the temporary hold of activity during the ongoing pandemic were generally favorable. Since the re-start of the program, transplant offers are only rarely rejected by patients (who are informed on SARS-CoV-2 and the unlikely chance of virus transmission and consent before the transplant). This may reflect the trust Austrian patients have in the decisions made by the transplant team and are in accordance with the broad acceptance of the intermittent but wide-reaching governmental regulations by the Austrian people.

As elsewhere [12], the living transplant programs were temporarily stopped in Austria. The percentage of living donation is traditionally low in Austria, accounting for an average of 16 percent of the total activity over the last three years. This differs significantly from other countries where living donation sometimes exceeds 50% of all transplants. Despite the relatively low numbers of living donations, the waiting list in Austria has remained stable over the last years/decade, owing to the high number of deceased donor organs transplanted in the country (40.9 per million population in 2017) [28]. The number of actively wait-listed patients also did not increase from 30 June 2019 (*n*= 627) to 2020 (*n*= 595).

SARS-CoV-2 had a demonstrable impact on the post-transplant care of patients in Austria. Significantly less patients than in 2019 were seen in the outpatient clinics during the lockdown, as non-essential control visits were postponed in order to free resources for inpatients. At the time, when the prevalence of the virus was still unknown, it was considered equally important to limit the exposure of patients to a potential infectious environment (e.g., waiting rooms). We believe that by that measure we could contribute to limit the spread of the disease among the transplant population, as later also suggested by others [19,29]. Whether the delay of the visits did or will result in transplant-specific or general medical problems that went undetected in our patients during the cessation period remains to be evaluated.

Until 30 June, only ten of 5512 prevalent patients (Source: Austrian Dialysis and Transplant Registry) contracted COVID-19, representing 0.18% of the transplant population. This rate did not differ from the general Austrian population (0.2%, i.e., 17,776 positive cases by 30 June). Several reasons could have led to this favorable outcome. First, transplant patients are well informed about their increased infection risk by their physicians. In addition, risk groups were addressed early through widely distributed information by Austrian health authorities and instructed to be particularly careful. Furthermore, the timely stay-at-home order imposed during the lockdown of the country may have been crucial, specifically for the protection of transplant patients.

The case fatality rate in Austrian kidney transplant patients (10%; only one patient death due to COVID-19 pneumonia) was much lower than in other series of transplant patients with COVID-19 [4]. Likely reasons are an easy and early access to hospital care for transplant patients in Austria and that our patients may have contracted a less severe disease leading to only two ICU admissions. These data are in line with the generally low COVID-19 case fatality rate in Austria (2.4%), which is significantly less than in other countries, as shown by the updated data reported by the Johns Hopkins University [3] (https://coronavirus.jhu.edu/data/mortality, accessed on 12 September 2020).

Data on the total number of Austrian kidney transplant patients who were tested for SARS-CoV-2 are lacking. Due to the national “stay-at-home” orders proclaimed by the government and the reduction in outpatient visits, it cannot be ruled out that some patients with mild symptoms did not report to their respective transplant center and remained undetected infected cases. Thus, it will be interesting to perform widescale SARS-CoV-2 antibody testing in the group of kidney transplant recipients in the future.

Taken together, our observations suggest that the effect of SARS-CoV-2 on the Austrian transplant activity was mitigated and could be contained within acceptable limits. The timely actions by the government and health authorities, which were based on scientific advice, and strategies instituted by hospitals and transplant centers were important contributors, as was the active contribution of the patients and their families, who complied with the recommendations. These observations are an important lesson that shows for the well-defined field of kidney transplantation how public measures can be of direct benefit for individual patient groups. The observations may be extrapolated to other specialized fields of medicine. Furthermore, the Austrian experience may serve as an example—at least for kidney transplant patients—of how the pandemic could be successfully navigated, at least in regions with similar population densities and transplant activities [30]. At the same time, we appreciate that it may be difficult to obtain the same results in other countries with different preconditions.

The importance of combined general and specific actions, integrating science and policy, should be respected by governments and healthcare providers in preparation for impending threats caused by a significant re-emergence of SARS-CoV-2 or other future deadly viruses.

## 5. Limitations

The study describes the influence of COVID-19 on kidney transplant activity in Austria and the effect of SARS-CoV-2 on Austrian transplant patients. While 10 patients were identified as SARS-CoV-2-positive during the study, data on the number of negative tests in Austrian kidney transplant patients are not available.

Our results demonstrate favorable transplant results. Whether the delay of outpatient visits did or will result in transplant-specific or general medical problems that went undetected in our patients during the cessation period remains to be evaluated.

The Austrian experience may serve as an example—at least for kidney transplant patients—of how the pandemic could be successfully navigated. Whether this strategy is equally effective in regions or countries with different population densities and transplant activities remains to be elucidated.

Due to the lack of other complete country data, we were not yet able to compare our findings with transplant populations of other nations that followed divergent strategies to fight the pandemic.

## Figures and Tables

**Table 1:** Timeline of measures implemented by the Austrian government during the course of concurrent SARS-CoV-2 infections (adapted from https://www.sozialministerium.at/Informationen-zum-Coronavirus/Coronavirus---Rechtliches.html, accessed on 7 August 2020).

	COVID-19 Relevant Dates in Austria
25 February	First two cases of SARS-CoV-2 in Innsbruck
27 February	First case of SARS-CoV-2 in Vienna
12 March	First COVID-19-associated death in Austria (at 302 recorded SARS-CoV-2-positive cases)
31 March	Peak (*n* = 1.110) of concurrently hospitalized COVID-19 patients
3 April	Peak (*n* = 9.193) of concurrently COVID-19 ill individuals
8 April	Peak (*n* = 267) of concurrently patients in intensive care (*n* = 267)
18 April	First day with less than 100 new SARS-CoV-2-positive cases
10 May	Less than 300 patients in hospital care
17 May	Less than 50 patients in intensive care
	**Measures imposed by the Austrian government**
10 March	Temporary ban of indoor events with >100 persons and outdoor events with >500 persons
11 March	Announcement of planned school and university closures (2,3 cases/100,000 population, 206 new cases recorded)
12 March	Universities close
15 March	COVID-19 Law passed (9 cases/100,000 population, 800 cases recorded)
16 March	Shops close (except grocery stores, pharmacies and drugstores)
	Restaurants allowed to open only until 3 p.m.
	Prohibition of visiting public places
17 March	All bars and restaurants close
18 March	All schools close
20 March	Home office publicly advised for non-essential work
1 April	Compulsory use of face covering/masks over nose/mouth in grocery stores, 1-m distance to be observed
14 April	Compulsory use of face covering/masks over nose and mouth for public transportation and in all shops
15 April	Re-opening of small shops
1 May	Re-opening of larger shops
15 May	Restaurants allowed to open with some restrictions
18 May	Low-graders allowed back to schools
25 May	Stepwise re-opening of schools
29 May	Re-opening of hotels
15 June	Use of face covering/masks over nose and mouth only for public transportation, in pharmacies and healthcare facilities and if 1 m distance is not feasible; restaurants allowed to open until 1 a.m.; easing of travel restrictions for most European countries

**Table 2:** Characteristics and outcomes of Austrian kidney transplant patients (*n* = 10) with SARS-CoV-2 infection between 28 February and 30 June 2020 (TAC—tacrolimus, MPA—mycophenoic acid, AZA—azathioprine, mTOR—mammalian target of rapamycin).

SARS-CoV-2 Infections in Austrian Transplant Patients		
Patient characteristics	Sex (male/female)	7/3
	Mean age (years; range)	53.1 (35–77)
	Ethnicity (Caucasian/Asian)	9/1
Reason for SARS-CoV-2 PCR test	Symptoms/screening	9/1
Transplantation	Type of transplant (deceased/living donation)	6/3
	Time since last kidney transplantation (years; range)	9.9 (1–20)
Comorbidity	Hypertension	7
	Coronary artery disease	2
	Diabetes mellitus	2
	Malignancy	1
	Obesity	2
Symptoms	Fever	6
	Cough	5
	Dyspnea	3
	Rhinitis	1
	Diarrhea	5
	Myalgia	1
Hemodynamic instability	(yes/no)	1/9
Oxygen saturation	(mean %; range)	95.5 (88–100)
Organ manifestation	Pneumonia	3
	Rise in creatinine	4
	ECG changes	1
	No organ manifestation	4
Immunosuppressive therapy	Triple/dual	8/2
	Steroid/TAC/MPA	4
	Steroid/TAC/AZA	3
	Steroid/mTOR/MPA	1
	Steroid/TAC	1
	TAC/MPA	1
	Recent T-cell-depleting therapy	0
Other therapeutics	Renin angiotensin inhibitor therapy	4
Clinical course	Hospital admission	8
	ICU admission	2
	Changes of immunosuppression (yes/no)	5/5
	Antiviral therapy	1
	Renal replacement therapy	0
	Days in hospital (mean; range)	14.1 (5–24)
Outcome	Death/alive	1/9
